# Outcome of Modified Pirogoff Amputation for Diabetic Foot Infection: A Single-Center Case Series

**DOI:** 10.7759/cureus.46156

**Published:** 2023-09-28

**Authors:** Tunku Naziha Tunku Zainudin, Maniventhan Nachimuthu, Mohammad Izani Ibrahim

**Affiliations:** 1 Department of Orthopedics and Traumatology, Kuala Lumpur Hospital, Kuala Lumpur, MYS; 2 Department of Orthopedics and Traumatology, Hospital Raja Permaisuri Bainun, Ipoh, MYS

**Keywords:** syme amputation, minor amputation, diabetic foot infection, modified pirogoff amputation, pirogoff amputation

## Abstract

Introduction: Diabetic-related foot condition is one of the most debilitating complications with a higher rate of failure in limb correction, reconstruction, or salvage surgery. Amputation is the final option after other surgical treatments have failed. Major amputation increases energy consumption, resulting in high dependency, decreased mobility, and poor prognosis. Consequently, minor amputation is preferred to resolve these problems but elevated wound complications leading to inadequate prosthesis fit, became a detriment to minor amputation. Strict selection of patients is crucial to ensure success and good functional outcomes as demonstrated in this retrospective study of this case series.

Methods*:* This case series included six patients who underwent Pirogoff amputation with the modification described by Nather and reported the procedure's outcome. The inclusion criteria for subjects were the presence of palpable posterior tibial artery (PTA) or at least biphasic Doppler signal and ankle-brachial systolic index (ABSI) more than 0.70. Other demographic data as well as hematological, inflammatory, and biochemical parameters that may affect wound healing such as Hb, HbA1c, ESR, CRP, WBC, and albumin were recorded as well. The rate and time for wound healing and bone union, presence of complications, and final ambulatory status of patients were determined as the outcome of this study.

Results: All of the patients had diabetic foot infections involving only the forefoot region with the presence of either palpable PTA or biphasic Doppler signal. Although the majority of the cases had deranged blood parameters, soft tissue and bone healing were achieved at variable times. Four had good outcomes as they were able to ambulate. One case was complicated with chronic wound dehiscence and another one had Pirogoff stump infection and required transtibial amputation.

Conclusion: With strict selection criteria, Pirogoff amputation may provide a good functional outcome with a lesser degree of complications compared to major amputation.

## Introduction

Diabetes mellitus is one of the most prevalent public health issues in the globe, and both industrialized and developing nations are experiencing an increase in prevalence over the decade [1,2]. The prevalence of type 2 diabetes mellitus worldwide was estimated to be 9.5% in a recent meta-analysis [3]. With an estimated 3.65 out of 21.71 million population (16.8%) adults affected by diabetes, Malaysia led the Western Pacific region in Southeast Asia (SEA) by a prevalence of 8.8% [4,5].

Whether minor or major, diabetic-related amputations have a significant failure rate. Minor, often referred to as distal amputation, is a surgical amputation carried out from the level of the ankle joint and below while major amputation is a procedure carried out above the ankle [6]. The goal of amputation is to preserve as much of the functional limb. This is due to the amount of energy required to move when walking with an amputated leg is inversely related to its length and number of remaining joints [7]. Major amputation has been linked to more expensive medical care, worse prognosis, and greater mortality rate [8].

Minor amputations like Chopart which is performed at the level of the transverse tarsal joint preserve the functional length of the extremity and provide a stable heel support and a broad base for walking. However, in Chopart amputation, the disadvantage of contracted Achilles tendon without an appropriate tendon transfer will cause painful equinus and varus contracture of the upper ankle, which discourages them from walking [9]. Syme amputation occurs at the level of malleoli; on the contrary has a 50%-88% higher wound healing success rate than transtibial amputation [9,10]. It offers a full weight-bearing surface, [11] and can assist diabetic patients with good posterior arterial circulation in walking [12]. The drawbacks of Syme amputation include a limb length discrepancy of approximately 4-5 cm, which can make walking problematic, high rates of heel pad migration, skin issues due to devascularization of the heel pad, and a significantly higher rate of limb re-amputation [12-14].

Pirogoff amputation was created back in 1854 by a Russian surgeon Nikolay Ivanovich in which the heel is kept and used as a support [10]. The original method described the plantar skin flap as left attached to the calcaneus, then rotated 90 degrees dorsally and fused to the raw tibia surface to create a sensate weight-bearing surface. This resulted in a limb length discrepancy of less than 5 cm. Kinner et al. described a few modifications from the original Pirogoff to improve outcomes and reduce the risks and complications [11]. This procedure has many advantages, including a stable tibia-calcaneum stump due to osseous healing, length preservation that requires less energy when walking, and ease for the patient to walk without a prosthesis because of fully weight-bearing stump and smaller limb length discrepancy [11-13]. Due to these advantages, our center is keen to feature this case series of six patients who underwent Pirogoff amputation with the modification described by Nather et al. [12] and reported on the outcome of the procedure.

## Materials and methods

This was a retrospective study of a single center in Hospital Raja Permaisuri Bainun, Ipoh, Perak, Malaysia. There were six patients subjected to Pirogoff amputation from July to October 2021. All patients were followed up for a mean range of 12-18 months. Three of them had recurrent infections at the previous ray amputation site, one had wet gangrene of the fourth toe, one case presented with infected diabetic foot ulcer up to the forefoot region, and one had forefoot gangrene. All of these cases had raised leukocyte and inflammatory markers which indicated active infection at presentation. All of the patients had poor diabetic control as well based on the glycated hemoglobin (HbA1c) taken during admission.

Pirogoff amputation was opted for these cases based on these inclusion criteria. On clinical assessment and radiographically, the infection was up to the forefoot region, both dorsalis pedis and especially posterior tibial artery (PTA) pulse was palpable or at least had biphasic signal on Doppler ultrasound, as well as the ankle-brachial systolic index (ABSI) of more than 0.7 [13]. Empirical intravenous (IV) Unasyn (ampicillin plus sulbactam) 3g TDS was started for all patients except for patient cases no 3 and 5 who were given IV Tazosin and clindamycin. It is based on National Antibiotic Guidelines until the culture sensitivity is obtained post-operatively and changed accordingly. 

All of the patients were consented for the surgical procedure. The surgery was performed by a single orthopedic surgeon with modification as described by Nather et al. [12]. After careful soft tissue excision and dissection, the talus is removed completely followed by transverse osteotomy of the distal tibia and both malleoli. To centralize the heel pad, the distal calcaneum is removed, and instead of a 90-degree osteotomy, a 60-degree oblique osteotomy is performed before fusing it to the bare distal tibia surface. Fusion is secured with two 6.5mm cannulated screws in a cross-fixation manner to achieve union and stability [12]. Wound inspection was performed on postoperative day 3, and suture to open (STO) was done on day 21 postoperative. All of the patients were referred for amputee rehabilitation and prosthesis fitting if feasible. Patients were allowed for weight bearing once both soft tissue and bone healing were achieved.

Hematological markers like hemoglobin and leukocyte, inflammatory markers which were C-reactive protein (CRP) and erythrocyte sedimentation rate (ESR), as well as biochemical markers especially glycemic monitoring, HbA1c, and albumin, were monitored and strictly optimized pre- and postoperatively to achieve good wound healing and to ensure total eradication of infection. Patients were discharged once the blood parameters showed improvement and were followed up regularly to observe the wound condition and healing.

In this case series, the demographic data of age, sex, and diagnosis were evaluated as evaluation criteria. In addition, clinical data such as the presence of palpable distal lower limb pulses (DPA and PTA) and Doppler ultrasound assessment with capillary refill time (CRT) were recorded. Blood parameters that indicated the severity of the infection, as well as factors that may have affected wound healing and the fate of the stump, were also evaluated. A positive outcome is defined as a patient who does not require below-knee amputation (BKA) and is independent in daily activities. The ability to ambulate with or without assistance is also viewed as a favorable outcome.

## Results

Based on the inclusion criteria mentioned in the case method, six patients were selected for Pirogoff amputation. The first case was a 45-year-old Malay gentleman with recently diagnosed type 2 diabetes mellitus. He presented with a week's history of left foot discoloration which he claimed was due to ill-fitting shoes. He sought treatment after the discoloration turned gangrenous with discharge and he had episodes of low-grade fever (Table 1). Upon clinical assessment, both DPA and PTA pulses were palpable with an ABSI of 1.07 (Table 2). His WBC was raised to 16.5 x10⁹/L, and so were the ESR 243 mm/hour and CRP 148.7mg/L. His glycemic control was decent (7.7%) with a good albumin level as well (32g/L) (Table 3). IV Unasyn was started empirically and an emergency procedure Pirogoff amputation was performed. Postoperatively, the wound site achieved healing in three weeks and the bone union was obtained at eight weeks. At the final follow-up, he was satisfied with the outcome as he could walk unassisted with a prosthesis and resume his work (Table 4).

**Table 1:** Demographic data of the cases that underwent Pirogoff amputation There was an equal number of males and females involved in this case series, with a range age from 45 years old up to 74 years old. Chinese were the majority race followed by Malay and Indian. All of the patients had type 2 diabetes mellitus.

No	Age	Gender	Ethenicity	Type of DM	Diagnosis
1	45	Male	Malay	2	Left wet forefoot gangrene. Area of erythema does not cross midfoot.
2	66	Female	Malay	2	Right infected wound post 2^nd^ Ray amputation site with big toe gangrene.
3	68	Male	Chinese	2	Infected left diabetic foot ulcer base of first MTPJ with exposed joint and pus discharged tracking to 2^nd^-4^th^ webspace.
4	60	Female	Chinese	2	Left wet gangrene 2^nd^ and 4^th^ toe with pus discharged from third webspace ascending proximal to base of 3^rd^ MTPJ.
5	74	Male	Indian	2	Right foot infected wound post Ray’s amputation first, second at third toe with 4^th^ toe wet gangrene.
6	63	Female	Chinese	2	Recurrent wound debridement post first and second right Ray’s amputation. 5^th^ toe dry gangrene with area of erythema at the junction of forefoot to midfoot.

**Table 2:** Clinical vascular assessment of the patients All of the patients involved in this study had at least biphasic signal upon PTA assessment with Doppler ultrasound, with two patients did not have palpable PTA pulse. While four patients had biphasic signal DPA and two had monophasic signal on assessment. Only two patients had palpable DPA pulse on clinical examination. Regardless, CRT of all toes evaluated were less than two seconds except for one and all of the patients had ABSI within range 0.84-1.12.

No	DPA assessment	PTA Assessment	Capillary refill time (CRT) SEC	ABSI
1	palpable, biphasic signal	palpable, triphasic signal	<2	1.07
2	not palpable, biphasic signal	palpable, biphasic signal	<2	0.93
3	palpable, biphasic signal	palpable, triphasic signal	<2	1.12
4	not palpable, monophasic signal	palpable, biphasic signal	<2	0.84
5	not palpable, biphasic signal	not palpable, biphasic signal	<2	1.21
6	not palpable, monophasic signal	not palpable, biphasic signal	>2	0.86

**Table 3:** Hematological and biochemical assessment taken during admission All of the cases presented had leukocytosis and raised inflammatory markers, i.e., ESR and CRP which indicate significant infection at presentation. All of them also had elevated Hba1c which showed poor three-month glycemic control as well as hypoalbuminemia.

No (reference range)	Hb (M14-18 F:12-16) (g/dL)	WBC (4.5-11.0) (x10⁹/L)	CRP (< 3) (mg/L)	ESR (M: 0-15 F: 0-20) (mm/H)	HbA1c (4-5.6) (%)	Albumin (34-54) (g/L)
1	14.2	16.5	148.7	243	7.7	32
2	10.1	17.3	164	182	10.4	28
3	12	20.3	378	219	8.5	20
4	13.8	14.7	169	264	8.2	27
5	9.2	25.1	252.3	362	12.8	24
6	9.3	11.8	87	113	11.9	26

**Table 4:** Operative and final functional outcome for the cases Patients were seen at two weekly intervals until wound healed. Subsequently all of them were reviewed and assessed every two months until they have satisfied with the final outcome and subsequently discharged from follow up at 18 months. All of them were referred to rehabilitation amputee program for stump care, prosthesis fitting and gait training post operatively. Two out of six patients had wound healed at three weeks while one patient had wound healing occurred at four weeks. While others had delayed wound healing at 12 and 28 weeks, respectively. Four patients had good outcome as they were able to ambulate on the Pirogoff stump on a short distance or with prosthesis and walking aid. The bony union of the stump was also achieved between eight to 16 weeks. One case had chronic discharge with delayed bony union but eventually achieved union and wound healing after removal of screws. Unfortunately, one case complicated with recurrent wound dehiscence and another case had non-healing wound which subsequently became infected and had to underwent transtibial (BKA) amputation. These two cases were exception whereby follow-up was extended up to 18 months.

No	Duration wound healing (weeks)	Duration bone union (weeks)	Complication	Further intervention	Ambulation status	Final outcome at 18 months
1	3	8	None	None	With prosthesis	Good
2	4	12	None	None	Stump short distance	Good
3	3	10	None	None	Walking frame with prosthesis	Good
4	20	16	Chronic discharge	Removal of screws	Stump with prosthesis	Good
5	28	20	Recurrent wound dehiscence	Non weight bearing ambulation	Wheelchair	Fail
6	Non healing	Non union	Infected stump	Below knee amputation	Wheelchair	Fail

The second case was a 66-year-old elderly Malay lady with a 13-year history of type 2 diabetes mellitus. She complained of right big toe blackish discoloration with pus discharge tracking proximally from the previous right second toe ray amputation (Table 1). Her PTA pulse was palpable while DPA flow was biphasic from Doppler. The ABSI recorded 0.93 (Table 2). The infective and inflammatory markers were raised (WBC 17.3 x10⁹/L, ESR 182 mm/hour, CRP 164mg/L). She had poor glycemic control as evidenced by her HbA1c of 10.4% and low albumin level of 28g/L (Table 3). IV Unasyn was started for her, and emergency surgery was performed once she was optimized. The Pirogoff stump healed well at four weeks, but the bony union was only achieved at 12 weeks. She was satisfied during her final visit as she could ambulate on her stump for short trips (Table 4).

Our third case was a 68-year-old Chinese male with eight years onset of type 2 diabetes mellitus, presented with infected left diabetic foot ulcer base of first MTPJ with exposed joint and pus discharged tracking to second-fourth webspace (Table 1). His peripheral limb pulses, DPA, and PTA were palpable with an ABSI of 1.12 (Table 2). He appeared unwell and septic looking, and it was reflected in his blood parameters whereby the WBC was 20.3 x10⁹/L, ESR was 219mm/hour and CRP was 378mg/L (Table 3). Empirical broad-spectrum antibiotics treatment with IV Tazosin 4.5g QID and IV clindamycin 600mg TDS were given due to sepsis and to reduce the bacteremia load. He underwent surgery once his condition was stabilized. Fortunately, his wound achieved healing in three weeks with the union of the bone stump achieved in 10 weeks. At the final follow-up, he was able to mobilize using a walking frame and prosthesis (Table 4).

The fourth case was a 60-year-old Chinese lady, who also had chronic diabetes mellitus for 10 years, presented with left wet gangrene second and fourth toe with pus discharged from the third webspace ascending proximally to the base of the third MTPJ (Table 1). On further assessment, the PTA pulse was palpable with a biphasic Doppler signal while DPA flow was detected as monophasic. ABSI measurement was 0.84 (Table 2) and the blood parameters also showed raised WBC (14.7 x10⁹/L), ESR (264mm/hour), and CRP (169mg/L) (Table 3). IV Unasyn was prescribed empirically as well. She was also found to have hypoalbuminemia with an albumin level was 27g/L (Table 3). As she fulfilled the criteria Pirogoff amputation was performed. She had a bone union at 16 weeks; however, the soft tissue was complicated with chronic discharge. The wound achieved healing at 20 weeks after the decision was made for early removal of screws after bone stump union. Subsequently, she was able to ambulate on the stump without further complications and was discharged from follow-up at 18 months (Table 4).

Our fifth case was almost similar to the third as this 74-year-old Indian gentleman was presented in septic shock secondary to a right foot infected wound post-ray amputation first, second, and third toe with 4th toe wet gangrene (Table 1). The affected limb pulses were only detected via Doppler ultrasound with a biphasic signal, and the ABSI recorded was 1.21 (Table 2). His blood investigations showed anemia (Hb 9.2g/dL), leukocytosis (WBC 25.1), and raised inflammatory markers (ESR 362mm/hour, CRP 252.3mg/L). His glycemic control was also poor with HbA1c of 12.8% and a low albumin level (24g/L) (Table 3). IV Tazosin and clindamycin were started to reduce the bacteremia load while prepping him for surgery. Despite blood parameters optimization pre- and post-operative, it was complicated with chronic and prolonged wound dehiscence, and he was bound to his wheelchair. During the last follow-up at 28 weeks, there was minimal serious discharge still. Bone union was able to be achieved at 20 weeks without further complications (Table 4).

The sixth case in the series was a 63-year-old Chinese lady with recurrent infected wounds post debridement and ray amputation first and second toes. Clinically there was 5th toe dry gangrene with an area of erythema at the junction of forefoot to midfoot (Table 1). DPA pulse was detected monophasic while PTA was biphasic from Doppler assessment as clinically both were unpalpable (Table 2). She was also found to be anemic (Hb 9.3g/dL) with raised ESR (113mm/hour), CRP (87mg/L), and HbA1c (11.9%). She had slightly elevated WBC (11.8 x10⁹/L) and was found to have hypoalbuminemia as well (26g/L) (Table 3). IV Unasyn was opted as the empirical treatment as the rest of the case was not septic, and Pirogoff amputation was performed on her. However, the stump became infected, and we had to revise the amputation to the transtibial level to eradicate the source of the infection. Afterward, she achieved wound healing in three weeks and was only able to ambulate with a wheelchair due to poor effort tolerance (Table 4).

## Discussion

Minor amputation or amputation below the ankle is less commonly performed by surgeons due to the risk of wound complications. However, preserving the stump as distal as feasible results in significantly less energy consumed in walking than transtibial amputation, leading to patient mobility and independence [14,15]. Ankle disarticulation is a function-sparing articulation level that is largely employed in the treatment of diabetic foot infection and gangrene caused by peripheral vascular disease [13]. Syme amputation has been shown to have a better prognosis in cases of trauma [16]. In patients with diabetic foot infections, the results of Syme amputation have been inconsistent. It was hypothesized that the Pirogoff amputation would have a greater success rate than Syme's amputation in carefully selected cases [17,18]. Nather et al. found a 54% success rate (7/13 cases), which they attributed to the careful selection of prospective Pirogoff amputation recipients [19].

In this case series, patients were meticulously selected based on their vascular patency, tissue nutrition, and immune defense to reflect their post-operative healing capacity. The patency of the PTA was determined by palpation and Doppler ultrasonography, which required at least biphasic waveforms. A significant requirement for Pirogoff amputation is PTA patency [14,19]. An ankle-brachial index of greater than 0.7 is also required to sustain wound healing after amputation in diabetic patients. It was also described by Pinzur et al. [9] in which wound healing could be achieved with an ankle-brachial index of greater than 0.5 [9]. This was observed in our series where good stump healing was achieved except for two cases of failure, which could indicate poor vascular patency that contributes to poor healing. In addition to vascular inflow competence, tissue nutrition and immunocompetence play an important role in wound healing. The requirements of hemoglobin greater than 10g/dL, albumin level of 35g/dL, and total lymphocytes of 1,500 were chosen [9,16,20]. High protein and diabetic diets were prescribed to all patients to either increase or maintain albumin levels, iron supplements for hemoglobin production along antibiotic treatment based on the sensitivity of tissue cultures. All of these parameters were monitored during follow-up and were addressed accordingly to achieve the desired level of wound healing.

As mentioned before Pirogoff amputation had the benefits of good weight-bearing stump and length preservation. All patients with healed Pirogoff stumps were able to walk short distances without prosthesis aid. The heel pad healing and its sensation is preserved. Although Syme amputation provides a weight-bearing stump, it has a high incidence of heel pad instability, skin problems, and length discrepancies [16]. The original Pirogoff was introduced to reduce these complications and improve the outcome [17]. Unfortunately, there is a paucity of recent data regarding this technique, functional outcome, survival, and unknown complication rates [18]. During his participation in the Crimean War, world-renowned Russian surgeon Nikolai Ivanovich Pirogoff introduced the classic Pirogoff procedure, in which the heel pad is attached to the distal tibia using a portion of the os calcis to produce an end-bearing stump. However, the disadvantage of his approach was that the bone interface was stabilized with a long-lasting contact cast, which prevented early stump use [19]. Consequently, few adjustments were made to improve the outcome. Rijken and colleagues reported in 1995 the successful use of a modified Pirogoff amputation with the calcaneum cut approximately 50° parallel to the posterior facet, with a favorable prognosis in every case of severe traumatic foot injuries [20]. Similar to Boyd's amputation, this technique reduced Achilles tendon tension, resulting in a decreased tibiocalcaneum compressive force. In 2003, Taniguchi et al. modified the technique by cutting the calcaneum perpendicular to its axis and rotating the calcaneum anteriorly to the distal tibia by 90 degrees [21]. Fixation was achieved with anteriorly placed staples to surmount Achilles tendon tension and provide compression force to the tibiocalcaneum interface. Langerfeld et al. and Nather et al. [12,22] introduced a modification of calcaneum cut to 60-degree oblique osteotomy in 2011 and 2014, respectively. This osteotomy angle maintains optimal heel pad positioning for axial loading [22]. In addition, it permits extensive excision of the diseased forefoot and subsequent tightening of the Achilles tendon. They reported a successful conclusion [12,22]. We followed the most recent modification technique by Nather and Langefeld and modified the positioning of compression screws on the non-weight-bearing portion of the calcaneum to prevent wound breakdown due to pressure from the screws.

Our study is subjected to some limitations. Its limited sample size, which consisted of a single center may underestimate the generalizability of the results beyond the population under study. In addition, the retrospective nature of this cohort may further cause its limitations. The lack of standardization with many versions or modifications to the Pirogoff amputation technique, resulting in greater ambiguity and less consistent outcome, is the final factor.

## Conclusions

In conclusion, the modified Pirogoff amputation is preferable to other forms of minor amputation for diabetic foot infection. This is because it preserves the length of the stump, has minimal energy loss, and allows unassisted short-distance walking thereby enhancing the mobility and independence of the patient. Strict case inclusion criteria and patient selection, as well as pre- and post-operative optimization and maintenance, are required to ensure a successful and favorable outcome. At the conclusion of the 18-month follow-up period, 66% of the cases in this case series were determined to be successful. However, a larger cohort study is required to evaluate the best standardizable modification method that could provide the greatest functional outcome for patients.
